# Electrospun PCL Mats Modified with Magnetic Nanoparticles and Tannic Acid with Antibacterial and Possible Antiosteosarcoma Activity for Bone Tissue Engineering and Cancer Treatment

**DOI:** 10.1021/acsbiomaterials.5c00116

**Published:** 2025-06-26

**Authors:** Anna Hlukhaniuk, Małgorzata Świętek, Vitalii Patsula, Olga Janoušková, Antonín Brož, Marina Malić, Anna Kołodziej, Aleksandra Wesełucha-Birczyńska, Jiří Hodan, Miroslav Slouf, Waldemar Tokarz, Beata Zasońska, Lukáš Bystrianský, Milan Gryndler, Lucie Bačáková, Daniel Horák

**Affiliations:** † 86879 Institute of Macromolecular Chemistry, Czech Academy of Sciences, Heyrovského nám. 2, 162 06 Prague, Czech Republic; ‡ Charles University, Faculty of Science, Albertov 2038, 128 00 Prague, Czech Republic; § 48283 Jan Evangelista Purkyně University in Ústí nad Labem, Faculty of Science, Pasteurova 3544/1, 400 96 Ústí nad Labem, Czech Republic; ∥ Institute of Physiology, Czech Academy of Sciences, Vídeňská 1083, 142 00 Prague, Czech Republic; ⊥ 201871 Jagiellonian University, Faculty of Chemistry, Gronostajowa 2, 30-387 Krakow, Poland; # 49811 AGH University of Science and Technology, Faculty of Physics and Applied Computer Science, Mickiewicza 30, 30-059 Krakow, Poland

**Keywords:** nanocomposites, magnetic nanoparticles, antibacterial, tannic acid, fiber scaffolds, bone regeneration

## Abstract

Modifying scaffolds with agents that at the same time positively influence osteogenic cells and have a negative impact on cancerous growth, is a promising solution for patients with bone tissue defects following tumor excision. Such materials may not only boost tissue regeneration but also limit the risk of cancer reoccurrence. In our study, we developed novel bifunctional scaffolds containing magnetic nanoparticles grafted with PCL (MNP@PCL) and tannic acid (TA), which may be directed to support normal bone cells and suppress osteosarcoma cells. First, MNPs were postsynthetically surface-modified, by grafting poly­(ε-caprolactone) (PCL) from the surface via ring opening polymerization of ε-caprolactone, to provide their uniform distribution within the polymer matrix. Then, fiber mats containing a fixed amount of MNPs (2 wt %) and increasing content of TA (0, 1, 5, and 10 wt %) were prepared by electrospinning method. Both MNP@PCL and TA decreased polymer crystallinity. The interaction between the MNPs and TA significantly influenced the mat morphology, thermal properties, and initial hydrolytic performance. The most intensive TA release was observed mainly within first 6 h of incubation, and it was 3.5-fold higher (ca. 0.02 mg of TA/per mg of mat) for mfPCL@TA-10 compared to mfPCL@TA-5. Moreover, TA-containing magnetic mats suppressed the metabolic activity of osteosarcoma cells. They also demonstrated enhanced antimicrobial properties against the bacteria typically accompanying orthopedic complications, reducing the population of Gram-positive bacteria by more than 90% compared to the neat PCL mat. This proves the high potential of these materials for combining cancer treatment with bone tissue engineering.

## Graphical abstract

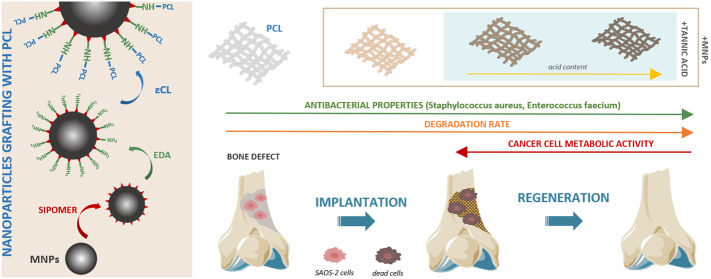

## Introduction

Although the incidence of primary bone cancer accounts for less than 1% of all oncology cases, almost all cancers metastasize to the bone, causing pain, fractures, spinal cord compression, and high blood calcium levels worsening patients’ quality of life.^1,2^ Complete removal of residual cancer cells and filling of bone defects after excision of the tumor are the major challenges for successful treatment of bone cancer. This is necessary to minimize the risk of the disease recurrence and metastasis and to provide the possibility of fast return to normal daily activity.^3^ Bone tissue engineering and bone regenerative medicine are rapidly developing areas, the main aim of which is to temporarily take over the function of damaged tissue and accelerate bone healing, allowing rapid recovery and restoration of full bone tissue functionality.^4^ Polymeric scaffolds are widely used as reparative materials due to their biocompatibility, good mechanical properties, and controlled biodegradation. Various natural, e.g., chitosan, gelatin, silk, collagen, and synthetic polymers, such as poly­(l-lactic acid), poly­(glycolic acid), poly­(ethylene glycol), and poly­(ε-caprolactone) (PCL), have been proposed for bone tissue engineering. PCL is a bioresorbable semicrystalline polyester, widely used not only in bone tissue engineering but also in drug delivery systems and wound dressings.^5^ Its advantages include satisfactory mechanical properties (high flexibility and superior strength), biocompatibility, cell growth stimulation, long degradation time, and permeability of bioactive agents. Most importantly, polymer matrices can be easily modified with additives, such as β-tricalcium phosphate, bioglass, hydroxyapatite, carbon nanotubes, magnetic nanoparticles (MNPs), cells, growth and stimulating factors, and drugs to boost both their physicochemical and biological properties.^6,7^ Phenolic compounds of natural origin seem to be particularly interesting modifiers of scaffolds intended for bone tissue applications, as in addition to antioxidant and anti-inflammatory properties, they can also promote osteoblastogenesis.^8–10^ Among the various phenolic compounds, tannic acid (TA) should be highlighted as it is not only a potent antioxidant, antimicrobial, antiviral, and anti-inflammatory agent, but it also upregulates bone formation markers, inhibits osteoclast activity, prevents osteoporosis, and cross-links collagen.^11–13^ Moreover, an antitumor and inhibitory effect of TA on a human osteosarcoma cell line has been reported.^14^ In addition, TA augmented the effect of cisplatin against human osteosarcoma cells (U2OS), suppressing cell proliferation and inducing apoptosis compared to chemotherapy alone.^15^ Modifying polymer matrix with more than one additive allows us to obtain bifunctional scaffolds capable of facilitating bone regeneration and providing anticancer activity.^16^ Such a multifunctional approach offers a solution to the existing limitations of traditional monotherapies.

Among the various scaffolds for bone tissue engineering, magnetic ones play a special role. To fabricate scaffolds with magnetic properties, iron oxide-based nanoparticles are commonly incorporated into the polymer matrix, as they are characterized by sufficient size-dependent magnetic properties and can be easily postsynthetically surface-functionalized. Moreover, iron oxide nanoparticles are known to be biodegraded intracellularly in lysosomes, which leads to the release of iron ions. Further participation of the iron in its natural metabolism makes these nanoparticles highly biocompatible.^17^ All these features enable the use of iron oxide nanoparticles in biomedical applications, such as magnetically assisted drug delivery, magnetic hyperthermia, and magnetic resonance imaging (MRI).^18^ Magnetic scaffolds can effectively contribute to the repair of defects and bone healing by magnetic therapy, which regulates cell activity (adhesion, proliferation, and differentiation) and accelerates the formation of new bone tissue.^19,20^ Another important advantage of magnetic scaffolds is that they facilitate the monitoring of tissue growth with common diagnostic techniques, such as magnetic resonance imaging (MRI).^21,22^ Magnetic guidance allows the possibility of overcoming physiological barriers and facilitates the controlled delivery of MNPs and active agents bound to their surface to the target site, which is particularly beneficial in transporting anticancer drugs to a tumor.^23^ Magnetic scaffolds used as a heat source can also sensitize tumor cells to cytotoxic drugs and can release cytostatics in a controlled manner.^24–26^ Recently, injectable biomimetic magnetic scaffolds were reported to collect drug-bearing nanoparticles to combine chemotherapy and magnetic hyperthermia to treat cancer, and concurrently provide the mechanical support required for bone regeneration.^27^

With this in mind, this research was focused on the design of bifunctional electrospun PCL-based mats modified with TA and superparamagnetic MNPs. It was expected that such mats will have microarchitecture and mechanical properties sufficient for mimicking bone tissue. TA was selected to endow the scaffolds with antibacterial and anticancer properties. The incorporation of MNPs was aimed to broaden the potential of the mats for both stimulating bone regeneration and anticancer treatment. To ensure uniform distribution of MNPs in the mats, PCL was surface-grafted onto iron oxide-based MNPs by ring opening polymerization of ε-caprolactone in a three-stage process. The amount of MNPs was fixed in all composites based on a previous study, where the cytotoxicity of magnetic nanocomposites increased with the increasing content of nanoparticles.^7^ Here, the effect of TA concentration on the fiber uniformity and size, thermal stability of the scaffold, its wettability, degradation in aqueous media, as well as cytotoxicity and antibacterial properties, was investigated.

## Materials and Methods

### Materials

4′,6-Diamino-2-phenyl-indole (DAPI), 0.9% sodium chloride solution, 25% ammonia solution, 4 Å molecular sieves, ε-caprolactone (CL), phalloidin-Atto 488, Dulbecco’s modified Eagle medium (DMEM), iron­(II) and iron­(III) chlorides, McCoy’s 5A medium, phosphate-buffered saline (PBS), poly­(ε-caprolactone) (PCL; *M*_n_ = 80,000 Da), sodium hydride 60% dispersion in mineral, and tannic acid (TA) were purchased from Sigma-Aldrich (St. Louis, MO). Chloroform, *N*,*N*-dimethylformamide (DMF), dichloromethane (DCM), diethyl ether, ethylenediamine (EDA), methanol, phosphorus pentoxide, tetrahydrofuran, and toluene were purchased from Lach-Ner (Neratovice, Czech Republic). Phosphate ester of poly­(propylene glycol monomethacrylate) (Sipomer PAM 200; SIPO; *M*_w_ = 451 Da) was supplied by Rhodia (Courbevoie, France). Alamar Blue cell viability assay and Gibco fetal bovine serum were bought from Thermo Fisher Scientific (Walthman, MA). Nutrient agar was purchased from Applichem (Darmstadt, Germany). MTS cell proliferation assay was purchased from Promega (Madison, WI). The ultrapure water used for the synthesis and modification of MNPs was produced by the Milli-Q IQ 7000 system (Merck Millipore; Burlington, MA). The CL was dried with calcium hydride and distilled prior to use. The tetrahydrofuran was dried with sodium hydride, distilled and stored over 4 Å molecular sieves. Chloroform was dried with phosphorus pentoxide, distilled and stored over 4 Å molecular sieves.

### Synthesis and Surface-Modification of MNPs

MNPs were synthesized by coprecipitating 0.2 M aqueous solutions of iron­(II) (50 mL) and iron­(III) chlorides (100 mL) with 0.5 M ammonia (100 mL) under sonication (Sonicator W-385; Heat Systems-Ultrasonics; Farmingdale, NY) for 5 min (40% amplitude).^28^ The resulting nanoparticles were separated using a magnet, washed with water until peptization, and redispersed in water. Synthesis of the nanoparticles was followed by their modification with PCL via a three-step procedure. First, MNPs (1.66 g; 0.007 mol) were mixed with SIPO (2.5 g; 0.005 mol) dissolved in DCM/toluene mixture (1:1 v/v) and stirred at room temperature (RT) for 4 h. MNP@SIPO particles were separated by centrifugation (1735 rcf) for 2 min, DCM was removed on a rotary evaporator (40–27 kPa) at 27 °C and the particles were washed with toluene and redispersed in toluene/methanol mixture (1:1 v/v; 50 mL). The amino groups were then introduced using the reaction between the methacrylic group of SIPO and EDA. For this step, MNP@SIPO particle dispersion was sonicated (30% amplitude) for 3 min, mixed with EDA (3 mL; 2.6 mol) and the mixture was stirred (700 rpm) at 40 °C for 96 h under Ar atmosphere. The resulting MNP@SIPO-NH_2_ particles were washed with diethyl ether (3 × 50 mL), dry tetrahydrofuran (3 × 12 mL), separated by centrifugation (3944 rcf) for 30 min and redispersed in dry chloroform (10 mL). Finally, PCL was grafted from the particle surface by ring-opening polymerization of CL via a reaction between its terminal hydroxyl groups and the amino groups of the modified nanoparticles. Suspension of MNP@SIPO-NH_2_ particles (60 mg) in chloroform was mixed with CL (2 g; 0.018 mol) under sonication (70% amplitude) for 5 min. After solvent removal on a rotatory evaporator (60 °C; 530 Pa), the mixture of particles and CL was polymerized at 160 °C for 18 h under Ar atmosphere. After cooling, the resulting MNP@PCL particles were purified by redispersion in DCM (20 mL) and precipitation in methanol (3 × 200 mL), dried under vacuum (530 Pa) for 4 h and stored under Ar atmosphere.

### Preparation of TA-Modified Magnetic PCL Nanocomposites (mPCL@TA)

Briefly, a 15 wt % PCL solution was prepared by dissolving PCL (1.066 g) in a DCM/DMF mixture (3:1 v/v) with stirring at RT overnight. MNP@PCL nanoparticles were dispersed in DCM (1 mL) under sonication (30% amplitude) for 3 min. In parallel, TA was dissolved in DMF (1 mL) in the dark under an Ar atmosphere. The solution was sonicated (amplitude 30%) with the MNP@PCL particle dispersion for 30 s, and the mixture was added to the PCL solution, which was degassed in an ultrasonic bath. The mixture was then transferred into a 2 mL syringe fitted with a 0.45 mm diameter needle. Neat PCL (fPCL) and magnetic mats (mfPCL) were produced using a home-built electrospinning device with a fixed needle-to-collector distance (15 cm), a constant infusion rate (750 μL/h) and voltage (13 kV). A neat fPCL mat and a mat containing 10 wt % of TA were prepared accordingly and used as controls. The composition of all produced electrospun mats is given in Table 1.

**Table 1:** Composition of the PCL-Based Mats

no.	denotation	content of MNPs (wt %)^a^	Content of TA (wt %)^a^
1	fPCL	0	0
2	fPCL@TA-10	0	10
3	mfPCL	2	0
4	mfPCL@TA-1	2	1
5	mfPCL@TA-5	2	5
6	mfPCL@TA-10	2	10

a Relative to PCL.

### Physicochemical Characterization of MNPs and PCL Composites

The morphology of neat and modified MNPs was analyzed by transmission electron microscopy (TEM) using a Tecnai G2 Spirit microscope (FEI, Brno, Czech Republic). The number-average diameter (*D*_n_) and dispersity (*Đ*) were calculated from at least 300 particles measured in the ImageJ program (version 1.53). For the infrared spectroscopy, the particles were analyzed in a mixture with potassium bromide, while the PCL mats were measured using an ATR-FTIR technique. All measurements were performed on a Bruker IFS 55 FTIR spectrometer (Billerica, MA) equipped with a mercury cadmium telluride detector and a Specac MKII Golden Gate Single Reflection ATR system (Orpington, U.K.) with diamond crystal and angle of incidence 45°. The spectra were collected with a resolution of 4 cm^–1^ and 64 accumulations. Raman spectra were measured using two laser lines, 785 and 1064 nm. The inVia Raman spectrometer (Renishaw; Wotton-under-Edge, U.K.) equipped with a Leica microscope (Wetzlar, Germany) was used to collect spectra excited by a 785 nm laser. The laser was focused at 50× magnification of the objective. The Raman scattered radiation was dispersed by diffraction grating (1200 grooves/mm); a CCD camera served as a detector. A Nicolet NXR 9650 FT-Raman spectrometer (Thermo Fisher Scientific; Waltham, MA) with an InGaAs detector with a 1064 nm laser line (Nd:YAG) was used for measurements. WiRE v. 2.0 and 3.4 software supplied by Renishaw and OMNIC software were used to process the spectra, including the elimination of cosmic spikes, correction of baseline, and smoothing of the spectra. Thermogravimetric analysis (TGA) was performed in the air on a Pyris 1 thermogravimetric analyzer (PerkinElmer; Waltham, MA) in the temperature range 30–800 °C with a heating rate of 10 °C/min. The saturation magnetization of the particles was determined using a 7300 vibrating sample magnetometer (Cryotronics; Westerville, OH) at 295 K. The morphology of PCL mats was visualized by scanning electron microscopy (SEM) using a MAIA3 microscope (TESCAN; Brno, Czech Republic). The mean fiber diameter was determined from at least 300 measurements using the ImageJ program (version 1.53). The water contact angle of PCL mats was measured using the static sessile method and contour analysis on an OCA 15EC device (Dataphysics; Filderstadt, Germany). The final values were means ± standard deviation (SD) of 10 individual measurements. Tensile tests were performed on an Instron 6025/5800R universal testing machine (Instron Ltd., High Wycombe, U.K.) with a speed of 10 mm/min and a cell load of 100 N. ISO527–3/5 dumbbell-shaped specimens with a total length of 60 mm, width of the narrowed part of 3 mm, and a thickness of 0.1 mm were tested. The resulting values were means ± SD of 6 measurements.

To evaluate the initial hydrolytic performance of mats, they were immersed in distilled water (mat to water = 1/1.4 w/w) and incubated at 37 °C for 50 days. The pH and conductivity of the incubation medium were initially monitored every day and then once per week. Then, the incubation was continued for 718 day; SEM images were taken after 368 and 718 days of incubation.

To monitor TA release, TA-modified mats were incubated in water at 37 °C and under a protective atmosphere to avoid possible oxidation of TA. The absorbance of the incubation medium was measured in duplicate after 0.5, 2, 6, 24, 48, 72, 96, 168, and 240 h in the range from 200 to 500 nm using a Specord 250Plus UV spectrometer (Analytik Jena AG; Jena, Germany) against water. For each composite, the incubation was conducted in parallel for three pieces of similar weight. The mass of incubated pieces of mats was adjusted to obtain absorbance between 0.02 and 1 au as this range was covered by a calibration curve prepared by measuring a series of TA aqueous solutions of decreasing concentration (initial concentration: 0.010 mg/mL, diluting factor: 1.25). The TA concentration in the incubation medium was determined based on the absorbance at 276 nm and recalculated to obtain cumulative TA release (mg) per mg of the composite mat. The final value is the mean of results obtained for three separately incubated composite pieces. After the release test, the pieces of mats were weighed to monitor weight loss.

### Evaluation of PCL Composite Biocompatibility

The biocompatibility of PCL mats was determined from cytotoxicity tests on two types of cells, i.e., rat bone marrow mesenchymal stem cells (rMSCs) and human osteosarcoma cells SAOS-2. The rMSCs were kindly provided by the Institute of Experimental Medicine of the Czech Academy of Sciences (Dr. P. Jendelová). The cell isolations were performed in accordance with the European Communities council directive of 22nd of September 2010 (2010/63/EU), follow the ARRIVE guidelines 1 and were approved by the Ethics Committee of the Institute of Experimental Medicine CAS, Prague, Czechia. Approval ID is 7848/2022. Then, cells were cultivated in DMEM supplemented with FBS, penicillin (100 units), and streptomycin (100 μg/mL) at 37 °C in an air atmosphere with 5% CO_2_. The rMSCs (1 × 10^5^ cells per mL) were subsequently incubated with PCL mats (0.8 cm × 0.8 cm) for 72 h in a 24-well flat-bottom plate (Techno Plastic Products; Trasadingen, Switzerland). After removing the mats, rMSCs were treated with Alamar Blue cell viability reagent (50 μL) for 4 h and the absorbance of resorufin was measured at 560 nm using a GloMax Explorer multiwell plate reader (Promera; Madison, WI). The percentage of living cells was calculated relative to the nontreated cells (control).

Prior to investigating the cytotoxicity of the materials toward SAOS-2 cells, the PCL mats were disinfected with 70% ethanol for 10 min, were washed with PBS and were placed in a 24-well flat-bottom plate. SAOS-2 cells (10,000 cells per cm^2^) were incubated with mats in McCoy’s 5A medium supplemented with 15% FBS at 37 °C in a 5% CO_2_ air atmosphere for 1, 3, and 7 days. To determine the metabolic activity of the adhered cells, the mats were transferred to fresh culture plates and incubated at 37 °C for 2 h in a 5% CO_2_ air atmosphere and with an MTS working solution prepared by mixing the MTS reagent with DMEM supplemented with 10% FBS in a 1:6 (v/v) ratio. The absorbance of MTS working solution was measured in triplicate at 490 nm using a VersaMax microplate reader (Molecular Devices; San Jose, CA), while the absorption of the background was read at 650 nm.

The morphology of SAOS-2 cells adhered to mats was investigated using an Olympus IX71 inverted epifluorescence microscope (Tokyo, Japan) equipped with a 10× objective (N.A. = 0.3). Preparation of the cells for microscopic observations included their fixing with 4% paraformaldehyde solution in PBS and staining with DAPI and Atto 488-conjugated phalloidin, which allow imaging of the nuclei and actin cytoskeleton, respectively. Cell micrographs were taken using a DP80 camera (Olympus) and processed by ImageJ software. From each material, including the control tissue culture PS, nine nonoverlapping pictures of cell nuclei (DAPI staining) were taken for image analysis. Cell numbers were counted for each picture using the Stardist plugin for ImageJ—Fiji software.^29,30^ Numbers of cells were recalculated to cm^2^.

### Antibacterial Properties of mPCL@TA Mats

The antibacterial properties of the mats were tested against four bacterial strains, i.e., , , , and , according to the published protocol.^31^ and bacterial cultures were grown in liquid LB medium at 37 °C, while and were grown in LB medium at 25 °C for 72 and 24 h, respectively, under shaking. Before incubation with PCL mats, bacteria were separated by centrifugation, washed with 0.9% NaCl solution (pH 7), and diluted to a concentration of 10^5^ bacteria/mL. PCL mats (0.8 cm × 0.8 cm) were incubated with bacterial suspension (100 μL) supplemented with 0.9% NaCl for 20 h. The concentration of viable bacteria was determined on nutrient agar under vortexing. Populations of and were counted after their cultivation at 27 °C for 24 and 40 h, respectively. In the case of and strains, the number of bacteria was determined after their cultivation at 37 °C for 24 h. Bacterial colonies grown without PCL mats were used as a control.

### Statistical Analysis

In most of the methods used for characterization of the materials, the ANOVA test with Bonferroni (wettability and mechanical properties) or Tukey’s (antibacterial properties), or Holm–Sidak (cell number) *post hoc* tests were used to find statistically significant differences between mats. The statistical differences between cytotoxicities of the mats toward SAOS-2 osteoblast-like cells was determined using Sigma Plot software (Grafiti LLC; Palo Alto, CA) with Tukey’s *post hoc* test.

## Results and Discussion

### Characterization of Magnetic Nanoparticles

TEM, FTIR, TGA, and magnetic measurements were used to analyze the particle composition after each modification step. Coprecipitation is one of the most commonly used methods to synthesize iron oxide-based MNPs with superparamagnetic properties. The main limitation of this approach is the irregular shape of nanoparticles and their polydispersity, which is the origin of their nonuniform magnetic behavior.^32^ According to TEM, the MNPs were semispherical in shape with diameter *D*_n_ ≈ 10 nm and dispersity *Đ* ≈ 1.09 (Figure 1a).

**Figure 1:**
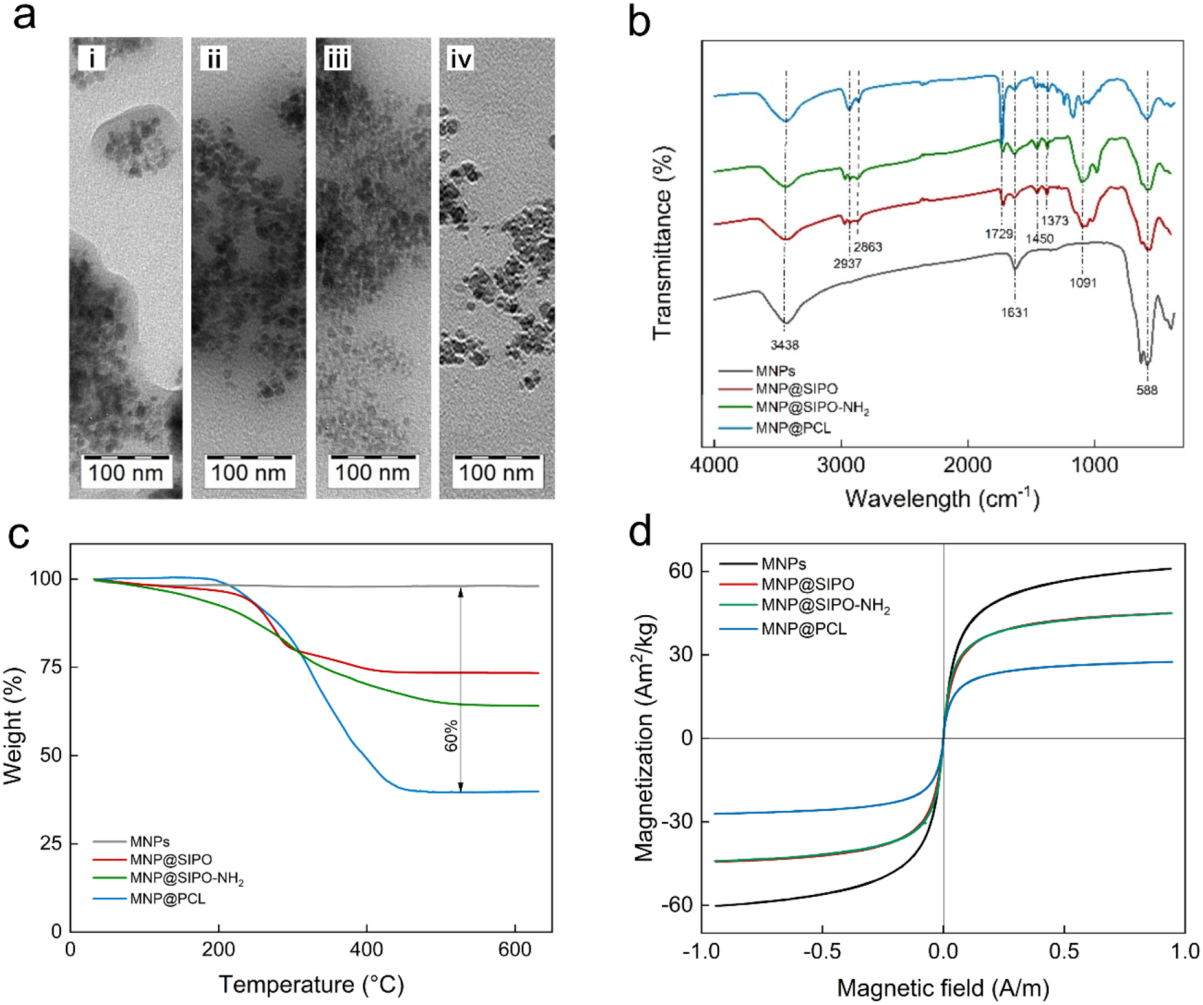
(a) TEM micrographs of (i) nonmodified MNPs, (ii) MNP@SIPO, (iii) MNP@SIPO-NH_2_, and (iv) MNP@PCL nanoparticles; (b) FTIR spectra; (c) thermogravimetric analysis; (d) magnetic properties of neat and modified nanoparticles.

The FTIR spectrum of neat MNPs showed typical stretching vibrations of the Fe–O bond, originating from the crystalline spinel lattice of iron oxide at ∼600 cm^–1^ (Figure 1b). A broad peak at 3463 cm^–1^ was assigned to the vibrations of −OH. Thermogravimetric analysis of MNPs showed a total weight loss of 2 wt % assigned to water (Figure 1c). The lack of magnetic remanence and zero coercivity indicated the superparamagnetic character of the MNPs; magnetic saturation reached *M*_s_ = 61 A·m^2^/kg and agreed with the literature data (Figure 1d).^33^

The TEM micrograph of the MNP@SIPO particles showed a thick layer of organic material around the particle cores that corresponded to the SIPO stabilizer (Figure 1a). In the FTIR spectrum of the MNP@SIPO particles, new peaks originating from SIPO appeared (Figure 1b). Peaks between 1153 and 1047 and 1452–1374 cm^–1^ were attributed to the stretching of ether C–O groups and the bending of CH_2_ moieties, respectively. C–C stretching was visible at 1637 cm^–1^, while a peak at 1722 cm^–1^ was assigned to carbonyl C=O vibrations. The typical antisymmetric stretching of CH_2_ groups appeared at 2973–2890 cm^–1^. According to TGA, the total weight loss for the MNP@SIPO was 27 wt %, of which 25% corresponded to SIPO (Figure 1c). This agreed with the 16 A·m^2^/kg decrease in the magnetic saturation of the particles (to 45 A·m^2^/kg) attributed to the presence of the nonmagnetic phase (Figure 1d).

Further modification of MNP@SIPO with EDA, introducing amino groups, slightly decreased the particle aggregation (Figure 1a). The successful modification of MNP@SIPO with amino groups was documented in the FTIR spectrum of the MNP@SIPO-NH_2_ particles by the appearance of a new peak at 1652–1735 cm^–1^ corresponding to amide I stretching (Figure 1b). Compared to the MNP@SIPO particles, amino-functionalization induced an additional 9 wt % weight loss according to TGA but had an insignificant effect on magnetic properties (Figure 1c,d).

The last step of MNP modification was the grafting of PCL. In the TEM micrograph, MNP@PCL particles showed a lower tendency to aggregate compared to other particles, most likely due to the effective steric repulsion by the thick PCL layer (Figure 1a). The FTIR spectrum was dominated by peaks typical for PCL, i.e., C–O–C stretching vibrations in the range of 1100–1200 cm^–1^, vibrations of C–O, C–C, and CH_2_ groups between 1200 and 1400 cm^–1^, stretching of C=O groups at 1727 cm^–1^, antisymmetric stretching of C–H bonds at 2952 cm^–1^ and OH stretching at 3438 cm^–1^ (Figure 1b).^34^ According to TGA, MNP@PCL particles were thermally stable up to 180 °C and then gradually degraded between 180 and 490 °C (Figure 1c). The total weight loss of MNP@PCL particles was 60 wt %, of which 24 wt % corresponded to PCL. The *M*_s_ of MNP@PCL was 27 A·m^2^/kg, which was 34 A·m^2^/kg lower than that for unmodified nanoparticles (Figure 1d). The content of the nonmagnetic phase determined from *M*_s_ was 44 wt % and agreed with the TGA results.

### Morphological and Physicochemical Characterization of Electrospun PCL Mats

Regarding the choice of scaffold fabrication technique, the decisive factor is the possibility of mimicking the target tissue. In particular, the intrinsic porosity mimicking natural bone tissue is important because it allows the integration of a large number of osteogenic cells and tissue growth.^35^ For tissue engineering applications, electrospinning has gained a lot of attention because it can provide hierarchically organized micro- and nanosized fibrous materials with three-dimensional interstitial spaces (pores) that stimulate the extracellular matrix. At the same time, such materials have good mechanical properties and a high surface-to-volume ratio, which is important for successful cell attachment, proliferation, and differentiation. The similarity of electrospun fibers to collagen fibers largely mimics the architecture of native bone tissue, which is an advantage over scaffolds prepared by other methods.

SEM microscopy was used to visualize the morphology of the electrospun PCL mats and to determine the average diameter of the polymer fibers (Figure 2). SEM micrographs showed smooth fibers without visible MNP aggregates in the case of magnetic PCL mats (mfPCL). Moreover, adhesive contacts between PCL fibers were observed, which could positively influence the mechanical properties.^36^ The nonmagnetic PCL fibers (fPCL) were the thinnest of all the materials produced with a mean diameter of 573 ± 242 nm (Figure 2a). Modification of the PCL matrix with MNP@PCL particles (mfPCL) doubled the fiber diameter to 1 μm and increased the polydispersity 1.7-fold (Figure 2b). No visible MNP aggregates in the mfPCL mats indicated improved distribution of MNPs within the polymer matrix or good compatibility of PCL-grafted nanoparticles, compared to previous reports.^7,37,38^ However, TEM observations of fibers would be required to confirm the lack of nanoparticle agglomeration at nano scale. An increased fiber diameter due to modification by different magnetic particles was previously reported and was attributed to the increased viscosity of the PCL solutions.^39^ However, our research showed a significant interplay between the amount of PCL grafted onto MNPs and the diameter of PCL fibers. Comparing composites with MNPs with different amounts of grafted PCL revealed that increasing PCL content led to the formation of thicker fibers.^40^

**Figure 2:**
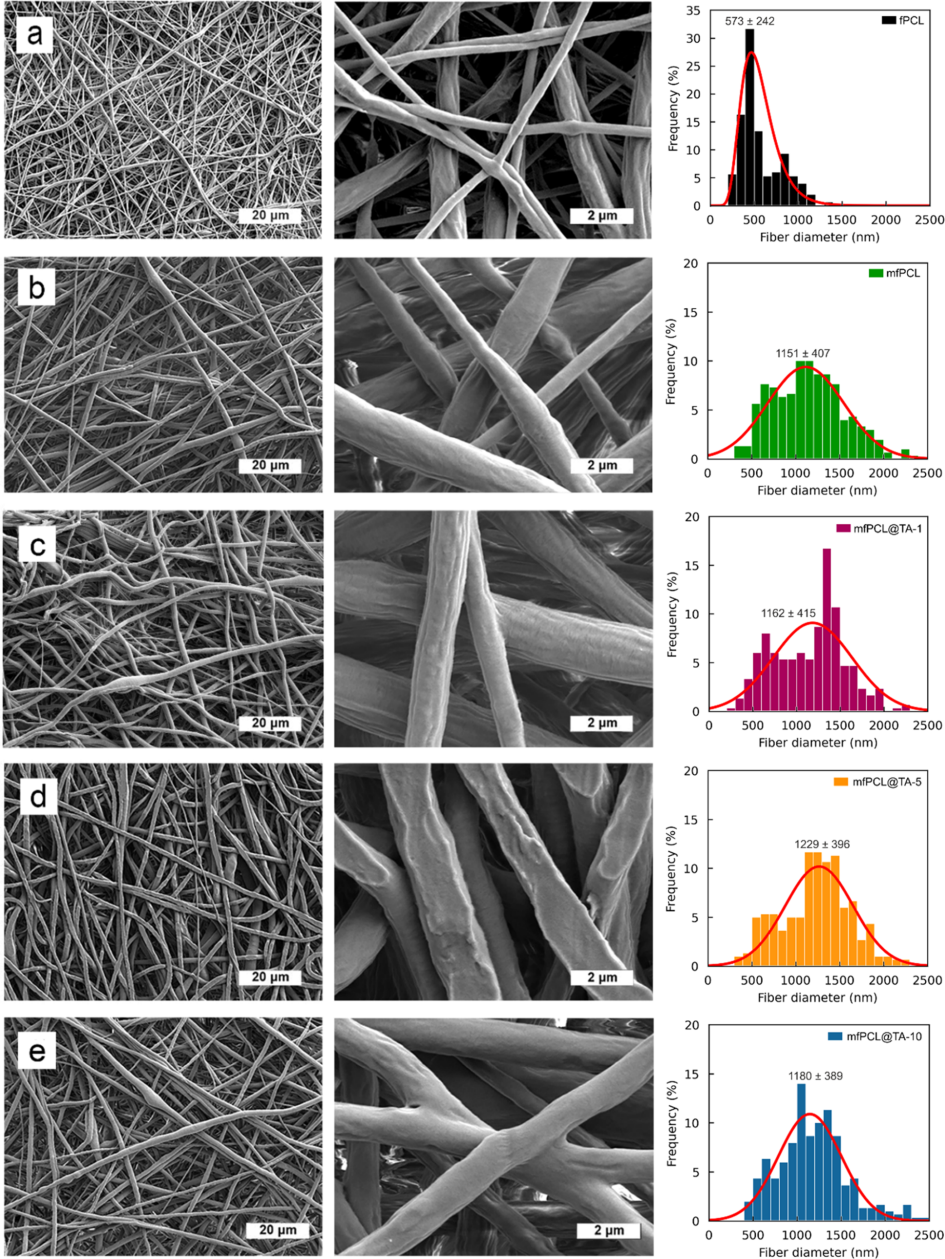
SEM micrographs and fiber diameter distribution of the PCL-based mats: (a) fPCL, (b) mfPCL, (c) mfPCL@TA-1, (d) mfPCL@TA-5 and (e) mfPCL@TA-10. Magnification 2000× (first column) and 20,000× (middle column).

In the magnetic PCL mats, TA did not have a significant effect on the fiber diameter and the polydispersity, irrespective of the amount of TA (Figure 2c–e). The fiber diameter in the mfPCL@TA-1 and mfPCL@TA-10 mats was almost the same (1162 ± 415 and 1180 ± 389 nm, respectively) and was also similar to those for mfPCL. The mat containing 5 wt % of TA (mfPCL@TA-5) showed slightly thicker fibers (1229 ± 396 nm), but the differences in fiber diameter were not statistically significant. Interestingly, modification of the PCL matrix with only TA increased the fiber diameter by 30% compared to fPCL (Supporting Information, Figure S1). This was most likely related to the reduced viscosity of the PCL solution, which caused stretching of the polymer jet during electrospinning, leading to the alignment of the PCL chains. However, our result contrasted with the PCL/TA composite obtained using 1,1,3,3,3-hexafluoroisopropanol acetic acid, for which the fiber diameter decreased with the increasing content of TA.^41^ This suggests a pivotal role of the solvent in affecting fiber diameter. Thus, it can be summarized that TA had a significant effect on PCL fiber diameter in the absence of MNPs, while the presence of MNP@PCL reduced the interactions between TA and the PCL matrix, minimizing the effect of the phenolic compound on the fiber size.

The ATR-FTIR spectra of all fabricated mats were dominated by peaks originating from the PCL. It should be emphasized that the MNP@PCL and PCL matrices showed very similar spectra. Bending, wagging, and stretching vibrations of methylene groups and gauche and trans isomerization of ester groups of PCL were observed in the range of 800–1300 cm^–1^, while peaks at 2958 and 2884 cm^–1^ were assigned to asymmetric and symmetric stretching of CH_2_ groups and a peak at 1727 cm^–1^ to C=O group of PCL (Figure 3a).^34^ From the TA-modified magnetic mats, only mfPCL@TA-5 and mfPCL@TA-10 showed a minor peak at ∼1610 cm^–1^. The same peak was also observed in the spectrum of the fPCL@TA-10 mat, originating from TA-related ν­(ring), δ­(OH) and δ­(CH) (Figure S2a).^42^

**Figure 3:**
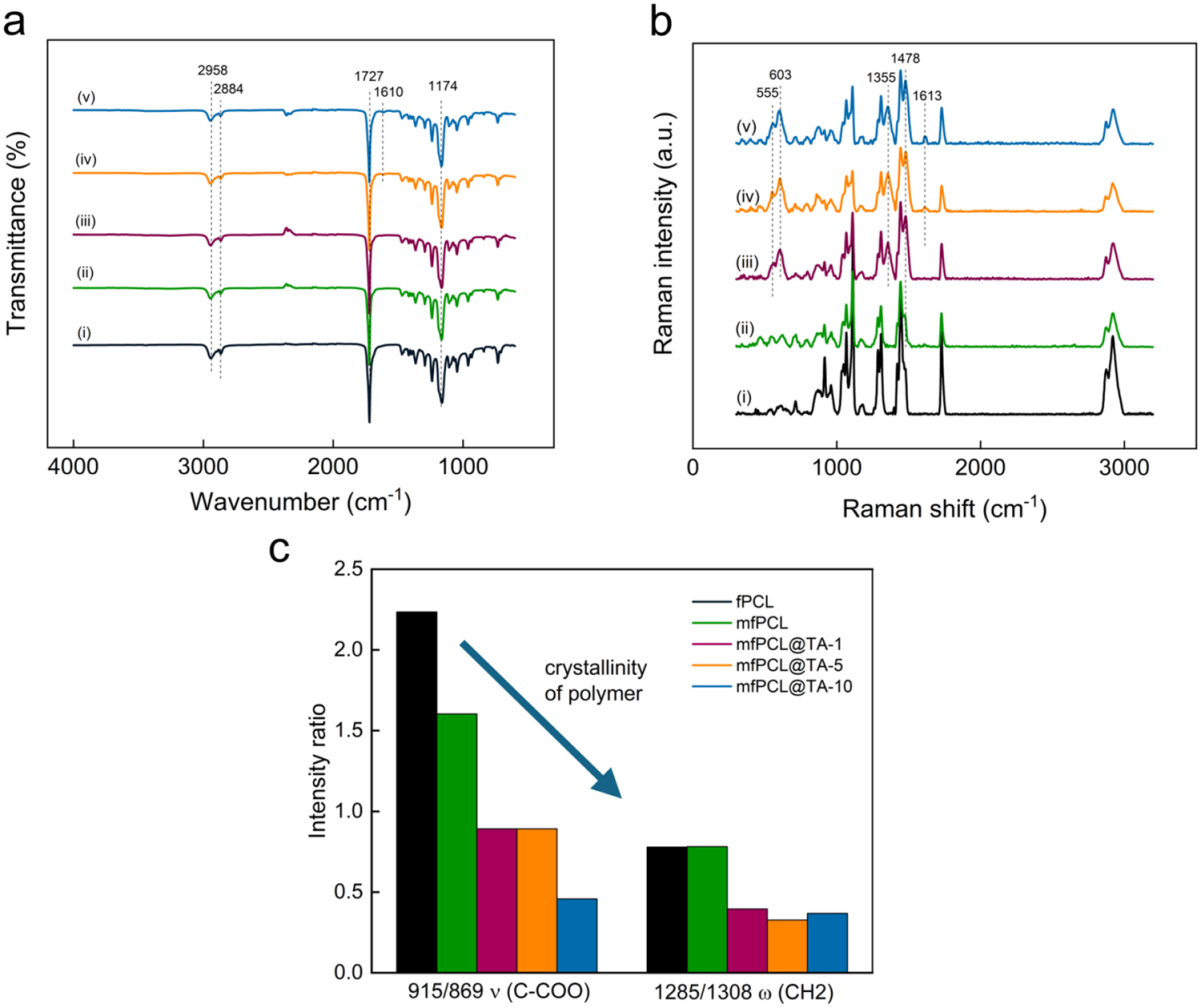
(a) ATR-FTIR and (b) Raman spectra of (i) fPCL, (ii) mfPCL, (iii) mfPCL@TA-1, (iv) mfPCL@TA-5 and (v) mfPCL@TA-10 mats. (c) Intensity ratios of crystalline bands to amorphous bands from vibration of the same molecule section.

In the Raman spectrum of the fPCL, characteristic bands of PCL were observed (Figure 3b and Table 2). Low band intensities were attributed to amorphous domains; the predominance of bands assigned to crystalline domains indicated the highly crystalline nature of PCL.^43,44^ Interestingly, no bands originating from MNP@PCL particles were detected in the spectrum of the mfPCL, and only a minor shift of the band attributed to the (CH_2_) cryst. vibration was observed from 1446 to 1443 cm^–1^, indicating that MNP@PCL particles disrupted the polymer crystallinity. The decrease in polymer crystallinity caused by particle incorporation was also confirmed by comparing the intensity ratios of the crystalline bands to the amorphous bands from the vibration of the same molecule section, i.e, 1285/1308 and 915/869 (Figure 3c). In contrast, clear new bands at 555, 603, 1355, and 1478 cm^–1^ were observed in the spectra of all mfPCL@TA mats. All these bands have been observed previously in iron gall and tannic inks, demonstrating the presence of the Fe-TA complex in PCL composites.^45,46^ The bands at 555 and 603 cm^–1^ were assigned to bidentate chelation of the Fe^3+^ ion by the phenolic oxygen of catechol.^47,48^ It is well-known that TA tends to form a stable complex with metals, employing catechol and/or galloyl groups.^47^ Depending on the pH, various numbers of TA molecules coordinate Fe^3+^, leading to the formation of mono­(catecholato)-Fe^III^ (pH < 2), bis­(catecholato)-Fe^III^ (3 < pH < 6), or tris­(catecholato)-Fe^III^ (pH > 7), which differ in color. While mono­(catecholato)-Fe^III^ is colorless and bis­(catecholato)-Fe^III^ is blue, tris­(catecholato)-Fe^III^ is brown. The darker beige hue of mfPCL@TA mats compared to mf@PCL and fPCL@TA mat indicated the presence of bis­(catecholate)-Fe­(III) or tris­(catecholate)-Fe­(III) complex in these composites (Figure S3). The exact attribution of the bands at 1355 and 1478 cm^–1^ is still under discussion; however, they likely originated from vibrations of the TA ring and hydrocarbon chain.^46^ Additionally, in the Raman spectra of mfPCL@TA-5 and mfPCL@TA-10 mats, a new band at 1,613 cm^–1^ appeared, which was also observed in the spectrum of fPCL@TA-10 mat (Figures 3b and S2b), indicating the presence of free TA. Free TA shows two dominant bands at 1613 and 1711 cm^–1^ assigned to the vibrational quadrant 8a stretching mode in the benzene ring and the stretching of C=O of the carboxylate, respectively.^48–50^ In a result of the interaction between TA and iron, the band at 1711 cm^–1^ disappears, regardless of whetever the measurement is performed for aqueous solutions or TA-Fe^III^ deposits. In turn, the decreased intensity and shift of the band at 1613 to 1580 cm^–1^ was observed only for aqueous solutions, while for TA-Fe^III^ it remained undetected. The interaction between MNP@PCL and TA proved that the polymer coating around the particles was not an impermeable layer, but it could be easily penetrated by the phenolic compound. Of note, PCL chains were not grafted directly from the nanoparticle surface but covalently bound via amino groups of the functionalized SIPO. An increasing number of SIPO molecules, chelating the iron exposed on the surface of MNPs, could thus increase the density of the polymer coating preventing further interaction with TA. Besides, the interaction between the core of MNP@PCL particles, the presence of nanoparticles may also influence the interaction between TA and PCL as the band attributed to ν (C–COO) vibration shifted from 867 to 870 cm^–1^ only in the case of mfPCL@TA mats. Moreover, H-bonding strong interactions between TA and polyesters, including PCL, were reported in the literature as a factor adversely affecting polymer crystallization by hindering the mobility of PCL chains.^41,51^ Indeed, with the increasing content of TA in the composite mats, the drop in polymer crystallinity can be observed (Figure 3c).

**Table 2:** Raman Shifts in cm^–1^ for PCL-Based Mats^a^

fPCL	TA	mfPCL	fPCL@TA-10	mfPCL@TA-X	assignment^42–44^
	362				δ (CO)
	548				
				555	ν Fe–O
				603	ν Fe–O (ν_3_)
	752				γCH + γOH
	780				
	833				γCH
867		867	867	870	ν (C–COO) *amorph*
916		914	915	914	ν (C–COO) *cryst*
959	954	962	960	960	ν (C–COO)
1043		1045	1042	1043	ν (COC)
1066		1066	1066	1066	ν (COC) *cryst*
	1090			1092	δ (CH)
1097*sh*		1096*sh*	1096*sh*	1096*sh*	ν (COC) *amorph*
	1197				δ (CH), δ (OH)
1111		1110	1111	1110	ν (COC) *cryst*
1285		1285	1286	1286	ω (CH_2_)
1308		1306	1307	1307	ω (CH_2_) *cryst* and *amorph*
	1335			1355	δ (CH), δ (OH), ν ring (ν_2_)
1420		1421	1420	1421	δ (CH_2_)
1446		1443	1443	1443	δ (CH_2_) *cryst*
1472		1471	1471		δ (CH_2_)
				1478	ν ring, δ (OH)
	1611		1613	1613	ν ring
	1712				ν (C=O) in ester
1728		1726	1727	1728	ν (C=O)
1737*sh*		1738*sh*	1739*sh*	1739*sh*	ν (C=O)
2873		2871	2871	2872	ν_sym_ (CH_2_)
2919		2919	2919	2920	ν_*as*_ (CH_2_)

a *X* = 1, 5, 10 wt % of TA, *sh*–shoulder.

All mats kept the superparamagnetic character of MNPs (Figure 4a). The *M*_s_ of mfPCL was 1.07 A·m^2^/kg, slightly lower (by 0.15 A·m^2^/kg) than theoretical predictions based on the *M*_s_ value of MNPs and the content of nanoparticles in the composite. Significantly, a negative effect of TA on the magnetic properties of mats was observed. While 1 wt % of TA did not influence the *M*_s_ compared to mfPCL, a gradual decrease by 0.02 and 0.11 A·m^2^/kg was recorded, with increasing content of TA for mfPCL@TA-5 and mfPCL@TA-10, respectively. This indicated that interactions between the magnetic core of the MNP@PCL nanoparticles and TA, confirmed also by Raman spectroscopy, led to partial surface oxidation of the particles and loss of their magnetic properties. However, the observed changes remained insignificant.

**Figure 4:**
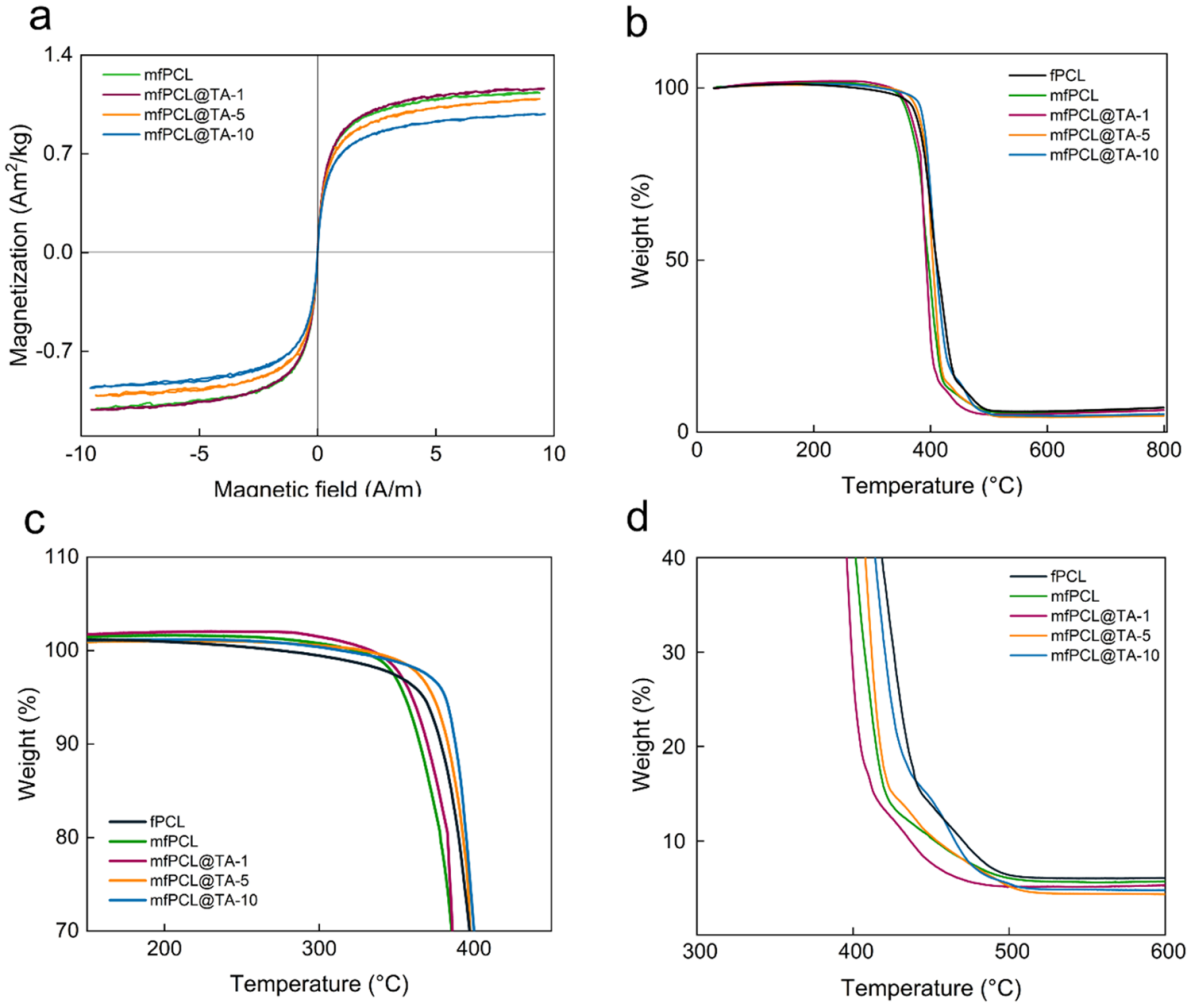
(a) Magnetic properties and (b–d) thermogravimetric analysis of PCL-based mats.

The thermal stability of the PCL mats was analyzed by TGA (Figure 4b). In all mats, initial weight loss (<5 wt %) ascribed to the removal of water was followed by the two-step degradation of PCL.^52^ The first stage, with onset below 400 °C, was assigned to the disintegration of ester chains accompanied by the release of water, carbon dioxide, and 5-hexanoic acid (Figure 4c).^53^ This process contributed to the main weight loss. In turn, the second degradation step occurring above 400 °C, was related to the depolymerization of PCL into ε-CL (Figure 4d). Modifying PCL only with the MNP@PCL shifted the onset of degradation to lower temperatures due to particle-induced random pyrolysis of the PCL chains (Figure 4b). The same effect was already reported by other authors for the PCL/Fe_3_O_4_ and poly­(l-lactic acid) modified with MgO.^38,54^ Compared to the mfPCL, the first step of PCL decomposition in the mfPCL@TA mats was gradually shifted to higher temperatures with the increasing content of TA, while the second one to lower temperatures. Of note, the initial weight loss of the fPCL@TA-10 had a bigger contribution to the total weight loss observed compared to magnetic composites, and the onset of the second degradation stage occurred at the highest temperature among all fabricated mats (Figure S4). The first observation indicated a higher capability of the fPCL@TA-10 mats to entrap water due to the hydrophilic nature of TA, while the second proved that TA delayed the thermal decomposition of the PCL matrix. This ability of natural phenols to form an intumescent char acting as a thermal barrier preventing polymer pyrolysis has already been reported.^41,55^ The significant differences in TGA between fPCL@TA-10 and mfPCL@TA-10 showed that the interactions between TA and the magnetic core of MNP@PCL particles strongly affected the water-binding capacity of TA (Figures 4b and S4). As a result, the interaction between TA and the polymer was no longer strong enough to prevent thermal degradation of PCL.

### Mechanical and Hydrolytic Properties of PCL Mats

The mechanical properties of scaffolds intended for tissue engineering are important due to the necessity of their good matching with the properties of body tissues. They strongly depend on both the chemical composition and the morphological features of the composites, including fiber diameter, uniformity, orientation, and density. Compared to scaffolds with parallel fibers, mats with randomly oriented fibers are in general characterized by lower packing density and bigger pore size distribution, which translates into poorer mechanical properties. However, all produced mats showed a random fiber orientation with no sign that either MNP@PCL or TA could influence this parameter (Figure 2). Three factors were expected to influence the most mechanical properties of magnetic composites compared to fPCL, i.e., distinct fiber diameter, the presence of the MNP@PCL, and the increasing content of TA. For neat PCL fibers, the rapidly decreasing Young’s modulus with increasing fiber diameter below specific fiber size has been reported in the literature, however, the reported “critical values” differ significantly, spanning from 80 to 1000 nm.^56^ Of note, there is a positive correlation between fiber size and porosity of the electrospun scaffolds.^57^ However, while the larger pores may facilitate the penetration of cells, they also deteriorate the mechanical properties. As the magnetic composites are characterized by ca. double-fold higher fiber diameter than fPCL, it can be expected that mainly this factor caused an observed drop in elastic modulus (Figure 5a,b). The presence of inorganic magnetic nanoparticles, characterized by significantly higher stiffness compared to PCL, was initially expected to improve the Young modulus as reported in the literature.^58,59^ The contradictory results observed for mfPCL can be attributed to the dominant morphological factor (fiber size) and low content of MNP@PCL in the magnetic composites. Furthermore, previous study showed that the amount of PCL grafted on the magnetic particles influences the mechanical properties of the mats, likely by determining the interfacial interactions between the particles and the polymer matrix.^40^ Even so, mfPCL were characterized by a significantly higher elastic modulus compared to PCL fibers containing 3 wt % of l-lysine-coated magnetite nanoparticles, proving the superiority of PCL grafting over l-lysine modification and indicating the pivotal role of the type of surface coating.^58^ While the literature suggested that neat PCL fibers had poorer mechanical properties than TA-modified ones, the effect of TA was insignificant for all mfPCL@TA mats.^41^

**Figure 5:**
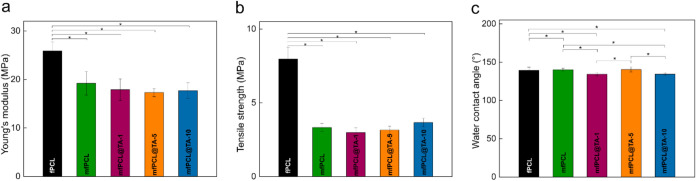
(a) Young’s modulus, (b) tensile strength, and (c) water contact angle of PCL-based mats. *, *p* < 0.05—statistically significant difference.

The wettability of the mat surface is influenced by various parameters, such as the type of polymer matrix, the morphology (roughness, size, and orientation of the fibers), and the surface chemistry, i.e., the nature and number of functional groups. All produced mats were hydrophobic in nature. Incorporating magnetic nanoparticles had almost no impact on the wettability compared to fPCL, for which the water contact angle (WCA) was 139° (Figure 5c). This observation was in contrast with our previous results and other authors, indicating that surface modification of MNPs, in particular its similarity to the polymer matrix, had a leading impact on the wettability of the mfPCL.^7,37^ Due to the strongly hydrophilic nature of TA, originating from its multiple catechol groups, it was expected that modifying PCL with this phenolic compound would decrease WCA. However, a small decrease in hydrophobicity was observed only in the mfPCL@TA-1 and mfPCL@TA-10 composites, where WCA dropped by 6° compared to mfPCL. This value, i.e., 134°, was significantly higher than the one reported for electrospun PCL-based fibers containing only 10 wt % of TA, for which WCA = 85°.^41^ Alternatively, treating Fe^3+^—modified PVDF membranes with TA led to a drop in WCA to an extent positively correlated with the increasing Fe^3+^ ion content. This indicated that the WCAs of the TA-modified mfPCL mats were the sums of the interactions between both TA and the PCL polymer matrix and TA and the MNP@PCL particles.

The initial hydrolytic performance of magnetic mats was investigated at 37 °C for 50 days by monitoring water pH and conductivity. Initially, up to day 43 of incubation, a deep pH decrease accompanied by an increase in conductivity was observed for all magnetic mats, indicating a cumulative effect of TA, PCL matrix and MNPs (Figure 6a). Although the decrease in pH positively correlated with the TA content and was the highest for the mfPCL@TA-10 mat, the conductivity results did not show the same tendency, suggesting that the product of hydrolytic degradation or impurities contribute more with time to the observed conductivity values. The differences registered on Day 1 in pH are mainly associated with the release of TA, which was the most intensive within the first 6 h of incubation (Figure 6a,c). These results agree with observations done by Chen et al., who also observed a rapid diffusion of TA from PCL-based electrospun mats.^41^ Only for mPCL@TA-10 mats, the release was prolonged and reached its maximum after 24 h, followed by the drop of TA concentration in the incubation medium. It indicates that, after initial release, TA can be later noncovalently rebound to the material. Noteworthy, the maximum amount of TA released from the mfPCL@TA-10 mats was almost 3.5-fold higher than the one observed for mfPCL@TA-5, which proved the weaker interaction of a significant part of incorporated TA and PCL or MNP@PCL particles. Of note, the released TA, although significantly influencing pH, had a minor effect on the conductivity. Weighting mats after the release test did not show changes bigger than the error of the balance used. The observed after 10 days cumulative release of TA corresponded to the ca. 8, 10, and 22% of incorporated into composite mats TA for mf@PCL-TA1, mf@PCL-TA-2, and mf@PCL@TA-10, respectively.

**Figure 6:**
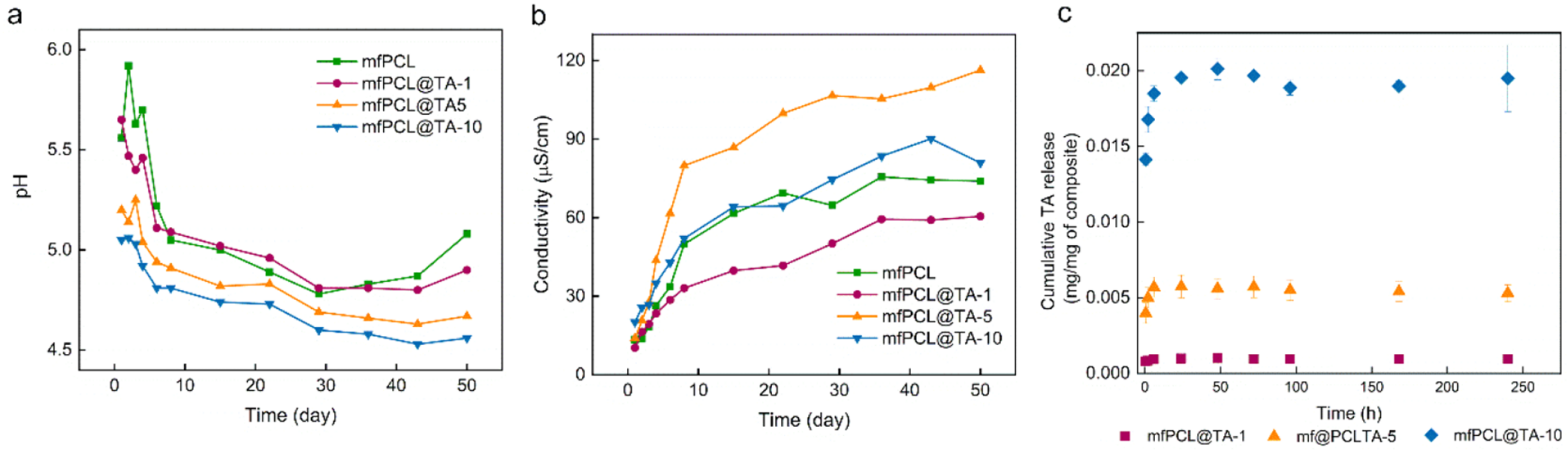
Dependence of (a) pH and (b) conductivity of water on the incubation time of PCL-based mats at 37 °C; (c) Cumulative TA release from mats incubated in water at 37 °C for 10 days.

Prolonged incubation (lasting 718 days) showed that the weight fraction of TA was the factor with a large impact on the hydrolytic performance of the mats and their long-term structural integrity (Figure S5). The TA-related negative impact of TA was also visible in SEM images taken after 368 and 718 days of incubation, showing tremendous changes in fiber shape (Figures S6–S7). Interestingly, mats without TA or with its low content was not uniformly wet due to the hydrophobic character of PCL. In general, PCL is a bioresorbable polyester characterized by longer degradation time compared to, e.g., poly-l-lactic acid and polyglycolide. The pH of the environment has a significant impact on the rate of PCL degradation; it has been shown that PCL degrades faster in an alkaline rather than an acidic environment.^60^ As the difference in pH between the incubation media of various composite mats in this report was not dramatic, it can be assumed that the release of tannic acid itself violates the integrity of the material, thus proving that TA binds (e.g., via hydrogen bonds) to multiple PCL chains stabilizing the fiber structure. Also, rebinding of released TA onto the surface of the fiber likely facilitates further wetting of the mats, accelerating its degradation. On the other hand, the long-term exposure of Fe^2+^ to oxygen likely caused the iron oxidation accompanied by the formation of superoxide, followed by the initiation of the Fenton reaction.^61^ As a result, powerful oxidizing agents, such as hydroxyl radicals, were generated. Of note, TA effectively mediates the conversion of Fe^3+^ into Fe^2+^, significantly accelerating the Fenton reaction.^62^ Despite the short lifetime of radicals, they may effectively scissor the polymer chains, leading to degradation and subsequent disintegration of PCL mats, particularly those with the highest TA content (mfPCL@TA-10).^63^ Moreover, the color change from beige to dark gray, which was observed for mfPCL@TA-5 and mfPCL@TA-10 mats, indicated the oxidation of TA followed by its decomposition or the presence of bis­(catecholate)-Fe­(III) complex in these composites (Figure S5).

### Cytotoxicity Test of PCL-Based Mats

To assess the ability of the TA added to the composite mfPCL mats to suppress the growth of osteosarcoma cells, the metabolic activity of the SAOS-2 cells using the MTS assay; nontreated cells seeded onto a PS plate served as a control (Figure 7). The results demonstrated that on day 1 after seeding, the metabolic activity of SAOS-2 cells was significantly higher in the TA-containing mats than in the control (Figure 7a). When comparing the composites with each other, the metabolic activity of cells on day 3 of cultivation significantly decreased with the increasing TA concentration (Figure 7b). However, the metabolic activity of cells on the mfPCL@TA10 mat remained the same as on day 1. Then, on the final day (day 7) of cultivation, the cellular metabolic activity was significantly higher on the mfPCL@TA-1 and mfPCL@TA-10 mats compared with the mfPCL@TA5 mat. The results on day 1 suggested that the presence of TA might improve the cell adhesion to mats, most likely by changing the surface energy and/or wettability. However, the results from day 3 clearly showed inhibitory effects of TA on osteosarcoma cell growth. In addition, the results on day 7 showed that the composite matrix was unlikely to provide the sustained release of TA required to completely suppress osteosarcoma cell growth (Figure 7c). The fluorescence microscopy images of SAOS-2 cells taken after 7 days of cultivation showed cells with normal morphology corresponding to the topography of the underlying microfiber mat (Figure 7d–g). In all time intervals, the SAOS-2 cells had a normally developed actin cytoskeleton. The micrographs showed a decreasing number of cells with increasing TA concentration in the mat, which was entirely consistent with the metabolic activity measurement. Thus, we can deduce that the MTS measurements correspond with the cell number and mainly measure cell proliferation over time. However, it must be highlighted that the measurement of metabolic activity does not provide an exact cell number or the cell population size. Other factors, e.g., the rate of cell metabolism, may influence the results achieved from this method. Furthermore, it has to be noted that the comparison of SAOS-2 cell number on PCL mats with the control tissue culture PS does not correspond with the metabolic activity result. The distribution of the cells on the mats was probably different compared to the tissue culture PS due to its different topography. The cells seeded on smooth PS can be carried, e.g., by convection currents appearing in the cultivation well before full attachment to the surface, whereas the cells seeded on fibrous mats nest on the surface right away. Considering the osteogenic nature of SAOS-2 osteosarcoma cells and the result of cellular metabolic activity on day 7, it can be speculated that TA, at least in a certain concentration range, has a positive effect on cell metabolism, which agrees with the literature data.^64–66^ However, it has to be mentioned that enhanced production of reactive oxygen species (ROS) in the long term, which is likely for mfPCL@TA-5 and mfPCL@TA-10 mats, can be a double sword of these composites.^67,68^ On the one hand, increased ROS production can be beneficial in terms of eliminating cancer cells, orchestrating the healing process, and providing a bacteria-free environment. Such strategies, adopting magnetic nanoparticles to promote the Fenton reaction and generate ROS, have already been proposed to induce the death of cancerous cells.^69,70^ On the other hand, ROS can also kill normal cells, affect their functioning (ROS are important mediators of signaling pathways), or destroy components of the extracellular matrix, leading to prolonged local inflammation that can impede tissue regeneration.

**Figure 7:**
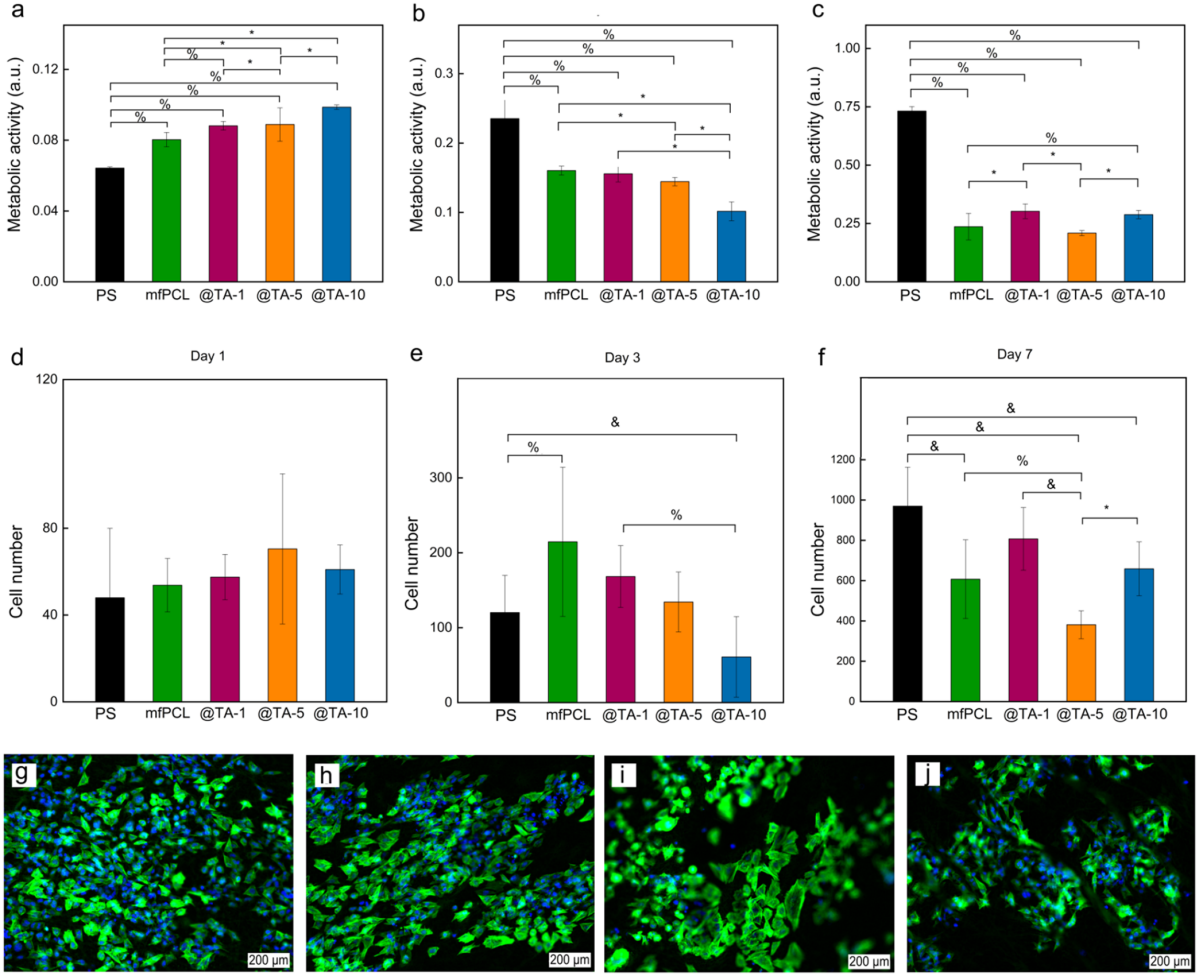
Metabolic activity and number of cells per cm^2^ of SAOS-2 cells cultivated on mats for (a, d) 1, (b, e) 3, and (c, f) 7 days; micrographs of cells attached on (g) mfPCL, (h) mfPCL@TA-1, (i) mfPCL@TA-5, and (j) mfPCL@TA-10 mats after 7 days. Control—PS—polystyrene plate (PS). %, *p* < 0.05 and *, *p* < 0.01, &, *p* < 0.001—statistically significant difference.

Bone marrow MSCs, also represented by rMSCc, are characterized by a high osteogenic potential, due to which they are widely used in bone tissue engineering and regenerative medicine, to also determine the ability of scaffolds to enhance and modulate the tissue repair process.^71,72^ In this study, only the viability of rMSCs seeded on PCL-based mats was assessed (Figure 8). Although the results did not reach statistical significance, both magnetic nanoparticles and the high content of TA showed a minor cytotoxic effect against rMSCc. Interestingly, rMSC cells incubated on the mats modified with particles in a similar way to MNP@PCL, but with a 30% higher content of grafted PCL, showed slightly higher viability.^39^

**Figure 8:**
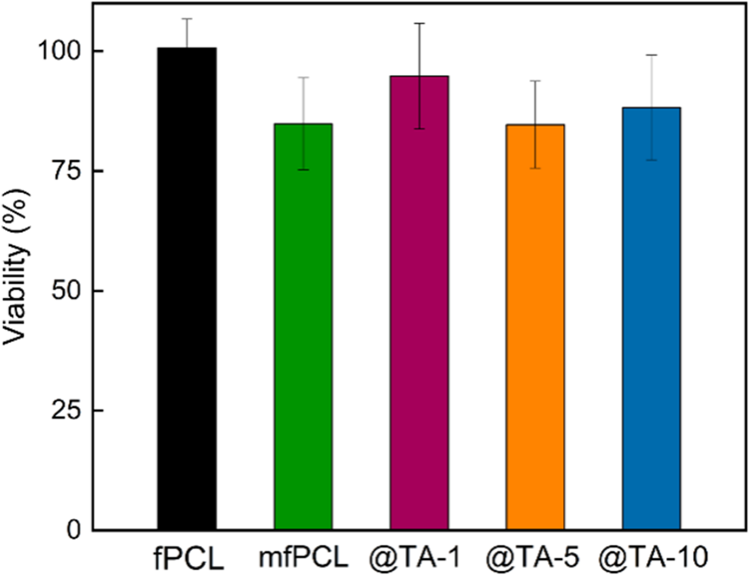
Viability of rMSC cells seeded for 72 h on PCL-based mats.

The effect of TA concentration, alone and in combination with iron oxide-based nanoparticles, on the rMSC response is worth further investigation, especially considering the fact that cell viability is strongly dependent on the level of ROS. TA-modified materials have been reported to increase the viability of bone marrow MSCs in inflammatory conditions by scavenging ROS or promoting their migration.^73,74^ However, in the presence of MNP@PCL, TA may contribute to the generation of ROS instead of their elimination, as mentioned above. This issue should be further clarified.

### Antibacterial Tests of PCL Mats

Thorough antimicrobial testing of PCL fiber mats was evaluated for , , , and bacteria (Figure 9). Infections caused by and are a typical cause of wound complications after orthopedic surgeries. Therefore, their effective prevention is essential to reduce morbidity and mortality, and improve patient’s quality of life. Although infections caused by and are rare in orthopedics, they can still induce osteomyelitis, which disrupts blood circulation in bone, leading to osteonecrosis. The response of the tested bacteria to the fPCL mat strongly depended on the thickness of their peptidoglycan layer in the cell wall; while Gram-positive bacteria and characterized by a thick peptidoglycan layer showed no statistical difference between control and fPCL mats, PCL strongly enhanced the colonization of Gram-negative bacteria (i.e., and ). No antibacterial activity of the fPCL mat was observed against and , which corresponds to the literature.^75^

**Figure 9:**
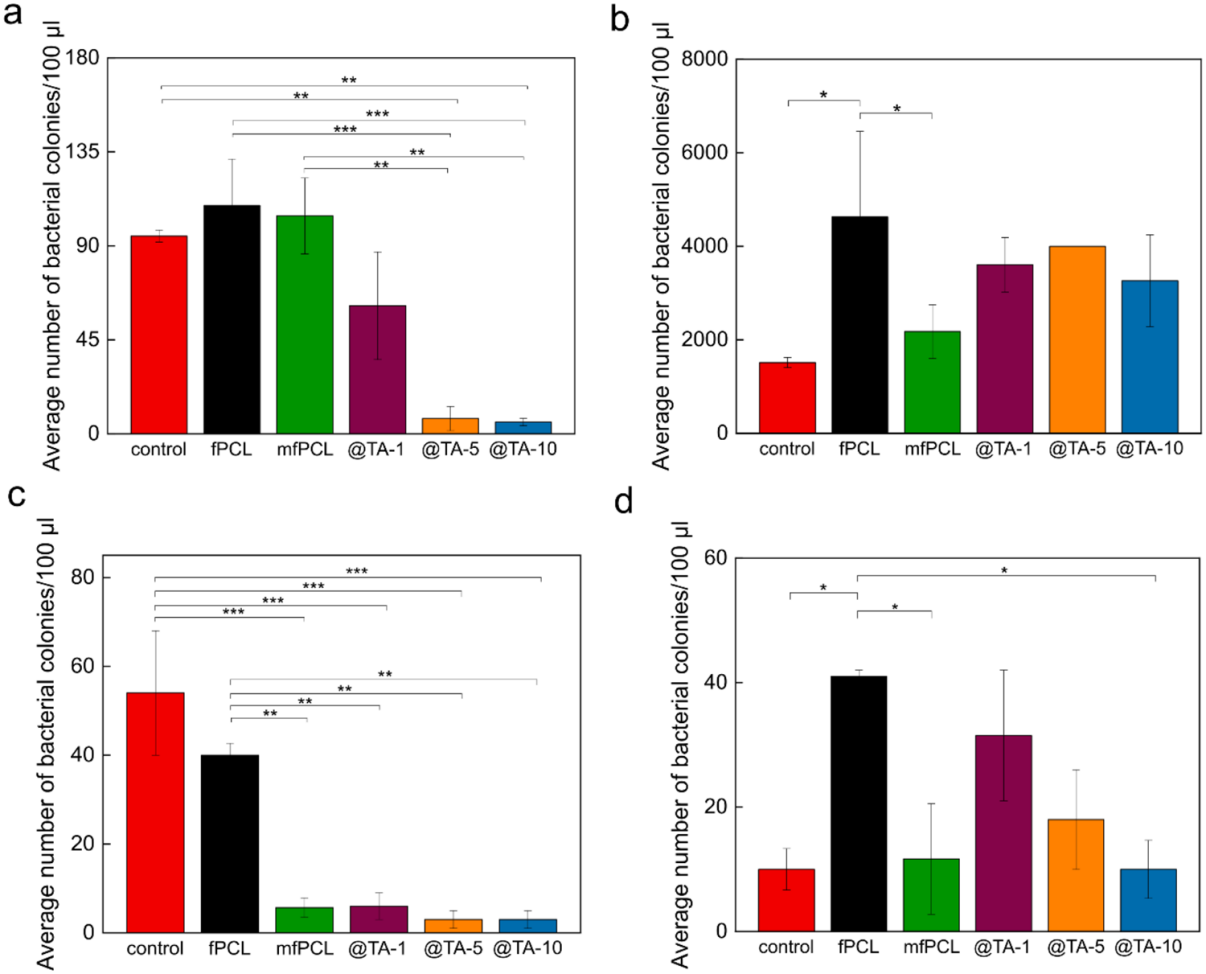
Population of (a) , (b) , (c) and (d) on different PCL-based mats. Control—bacteria in the absence of PCL mat. *, *p* < 0.1; **, *p* < 0.01—statistically significant difference.

Interestingly, modification of the PCL matrix with the MNP@PCL nanoparticles significantly suppressed colonization of the mats by most of the bacteria tested, except for (Figure 9). The antibacterial activity of MNPs against both Gram-positive and Gram-negative bacteria has been reported previously and stems both from the small particle size, which allows interaction with the cell wall, leading to disruption of plasma membrane permeability, and the ROS generated by MNPs inhibiting pathogenic bacteria. However, in the tests performed, the effect was not equally pronounced because the MNPs were incorporated into the PCL matrix, which may have limited the interaction of MNPs with bacteria.^76,77^

Further modification of mfPCL with TA reduced the number of colonies in a concentration-dependent manner, almost eradicating bacteria when, using mats containing 5 and 10 wt % of TA (Figure 9a). These mats also slightly reduced the number of colonies (Figure 9c); however, these results did not reach statistical significance. Similar TA activity against and was reported in the literature.^78^ Considering the disintegration of the mfPCL@TA-5 and mf@PCL@TA-10 mat, which was probably associated with ROS formation due to the Fenton reaction between TA and the MNPs@PCL particles, these two mats can be expected to be the most effective in eradicating bacteria in the long term via ROS-induced damage. In contrast, TA promoted the growth of colonies, which resulted from the high tolerability of *Klebsiella* strains to TA and their ability to degrade TA by tannase and to use the degradation products as a source of carbon and energy (Figure 9b).^79^

In the test with , the introduction of TA into the mfPCL mats at a low concentration resulted in a slight increase in the number of colonies compared to the mfPCL (Figure 9d). This was related to the supplementation of the bacteria with iron, caused by its chelation with TA, which led to the growth of .^80^ However, this effect was diminished with increasing TA content in the fiber mats. The demonstrated antibacterial activity in the presence of TA in fiber mats correlated with the reported activity of tannins showing a bacteriostatic effect rather than bactericidal activity.^79^ The nature of this behavior is due to the presence of many phenolic hydroxyls, which provide hydrophilicity and therefore form complexes with enzymes and cell wall proteins increasing membrane permeability. Another mechanism of antibacterial activity is the formation of complexes with metal ions that hinder their accessibility to bacteria. Comparing the results obtained, it was found that modification of fiber mats by the MNP@PCL particles and TA had a more pronounced influence on Gram-positive bacteria, which is due to the different structures of the bacteria cell envelope, in particular the presence of the bilayer membrane.^81^ Of note, the antibacterial properties of orthopedic implants containing components of natural origin are a promising strategy to challenge the growing problem of bacterial resistance to antibiotics.

## Conclusions

Various studies have shown that electrospun PCL scaffolds, which can be further modified for drug delivery, are a beneficial tool for guiding bone tissue regeneration. The TA release from mats increased with TA content and was mainly observed within the first 6 h. Released within 10 days TA corresponded to ca. 8, 10, and 22% of incorporated into composite mats TA for mats contains 1, 5, and 10 wt % of TA, respectively. Measurement of the cell metabolic activity of SAOS-2 cells cultivated on TA-containing PCL mats proved that TA shows not only a positive osteogenic effect, but can also suppress the osteosarcoma cells when it is delivered in a specific concentration, which makes TA-modified mats promising materials for bone tissue replacement in patients after tumor resection. Moreover, the mat combining 2 wt % of MNPs and 10 wt % of TA significantly enhanced the antibacterial properties against three bacterial strains, i.e., , , reducing their colonies by more than 90% compared to the mat made of pure PCL. It may limit the bacteria-related complications and positively influence the healing process. Furthermore, it was demonstrated that through changing the concentration of TA, the degradation time of mfPCL@TA mats can be controlled. As in this report we did not study the effect of combining the external magnetic field with magnetic scaffolds, this issue needs to be further investigated to completely assess the potential of magnetic TA-modified mats for bone tissue replacement.

## Supplementary Material



## Notes

Additional experimental data includes SEM photographs and fiber size distribution of fPCL@TA-10 mat, ATR-FTIR and Raman spectra, photographs of mfPCL@TA-1, mfPCL@TA-2 and mfPCL@TA-10 mats, TGA curve of fPCL@TA-10 mat, photographs of materials freshly removed from incubation after 718 days, and SEM images taken after 368 and 718 days of incubation (PDF)
