# Supplementary material for: Electrospun PCL Mats Modified with Magnetic Nanoparticles and Tannic Acid with Antibacterial and Possible Antiosteosarcoma Activity for Bone Tissue Engineering and Cancer Treatment
Source: ACS Biomater Sci Eng. 2025 Jun 26;11(7):4315–30. doi: 10.1021/acsbiomaterials.5c00116 (PMC12264861; doi:10.1021/acsbiomaterials.5c00116)
Supplement: Supplementary file 1 [file ab5c00116_si_001.pdf]

Supporting Information

Electrospun PCL mats modified with magnetic nanoparticles and tannic acid with antibacterial and possible anti-osteosarcoma activity for bone tissue engineering and cancer treatment

Anna Hlukhaniuk[a,b]*, Małgorzata Świętek*[a]*, Vitalii Patsula*[a]*, Olga Janoušková*[c]*, Antonín Brož*[d]*, Marina Malić*[d]*, Anna Kołodziej*[e]*, Aleksandra Wesełucha-Birczyńska*[e]*, Jiří Hodan*[a]*, Miroslav Slouf*[a]*, Waldemar Tokarz*[f]*, Beata Zasońska*[a]*, Lukáš Bystrianský*[c]*, Milan Gryndler*[c]*, Lucie Bačáková*[d]*, Daniel Horák*[a]

[a] Institute of Macromolecular Chemistry, Czech Academy of Sciences, Heyrovského nám. 2, 162 06 Prague, Czech Republic

[b] Charles University, Faculty of Science, Albertov 2038, 128 00 Prague, Czech Republic

[c] Jan Evangelista Purkyně University in Ústí nad Labem, Faculty of Science, Pasteurova 3544/1, 400 96 Ústí nad Labem, Czech Republic

[d] Institute of Physiology, Czech Academy of Sciences, Vídeňská 1083, 142 00 Prague, Czech Republic

[e] Jagiellonian University, Faculty of Chemistry, Gronostajowa 2, 30-387 Krakow, Poland

[f] AGH University of Science and Technology, Faculty of Physics and Applied Computer Science, Mickiewicza 30, 30-059 Krakow, Poland

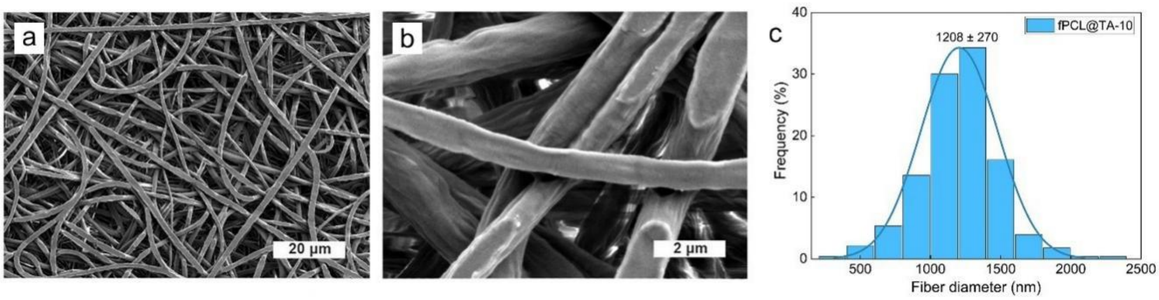

Figure S1. (a,b) SEM photographs and (c) fiber size distribution of fPCL@TA-10 mat Magnification 2,000 (first column) and 20,000 (middle column).

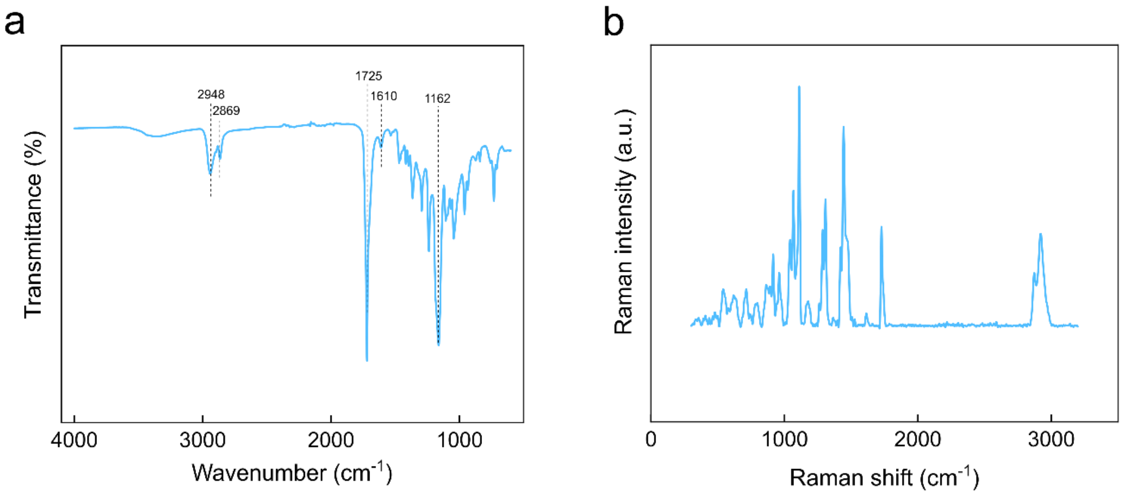

Figure S2. (a) ATR-FTIR and (b) Raman spectra of fPCL@TA-10.

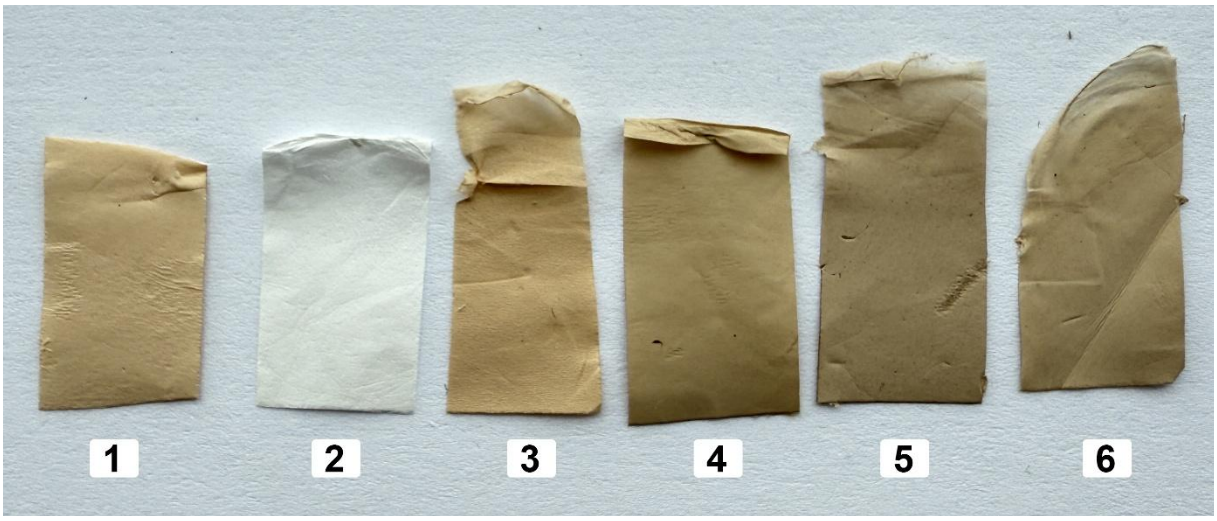

Figure S3. Produced PCL-based mats: (1) fPCL@TA-10, (2), fPCL, (3) mfPCL, (4) mfPCL@TA-1, (5) mfPCL@TA-2, and (6) mfPCL@TA-5.

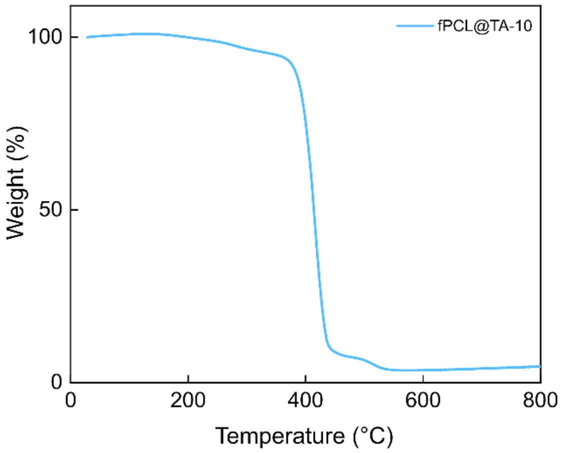

Figure S4. TGA of fPCL@TA-10.

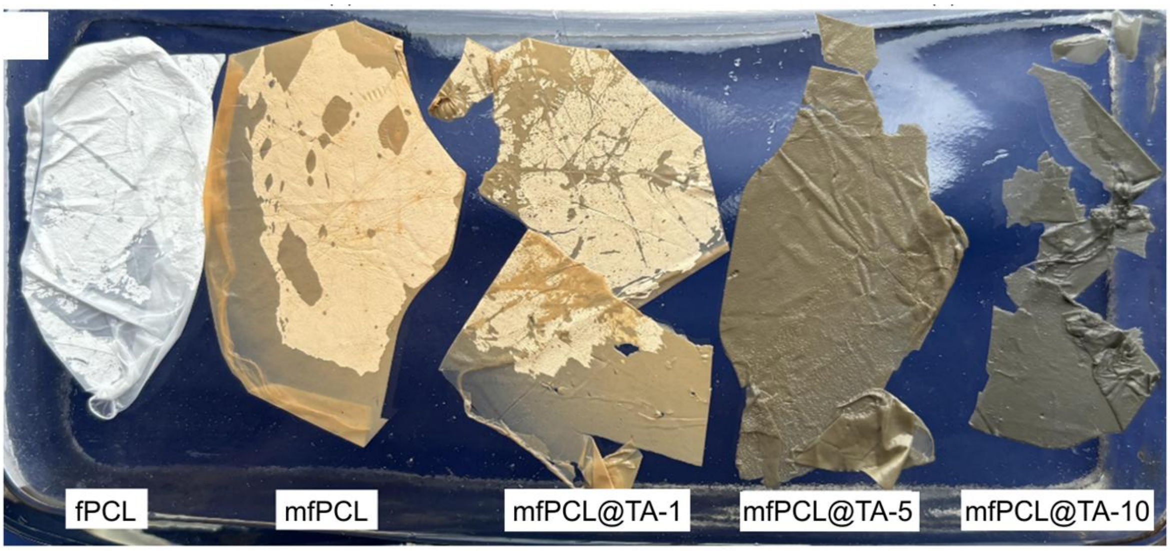

Figure S5. Fiber mats freshly removed from incubation lasting 718 days.

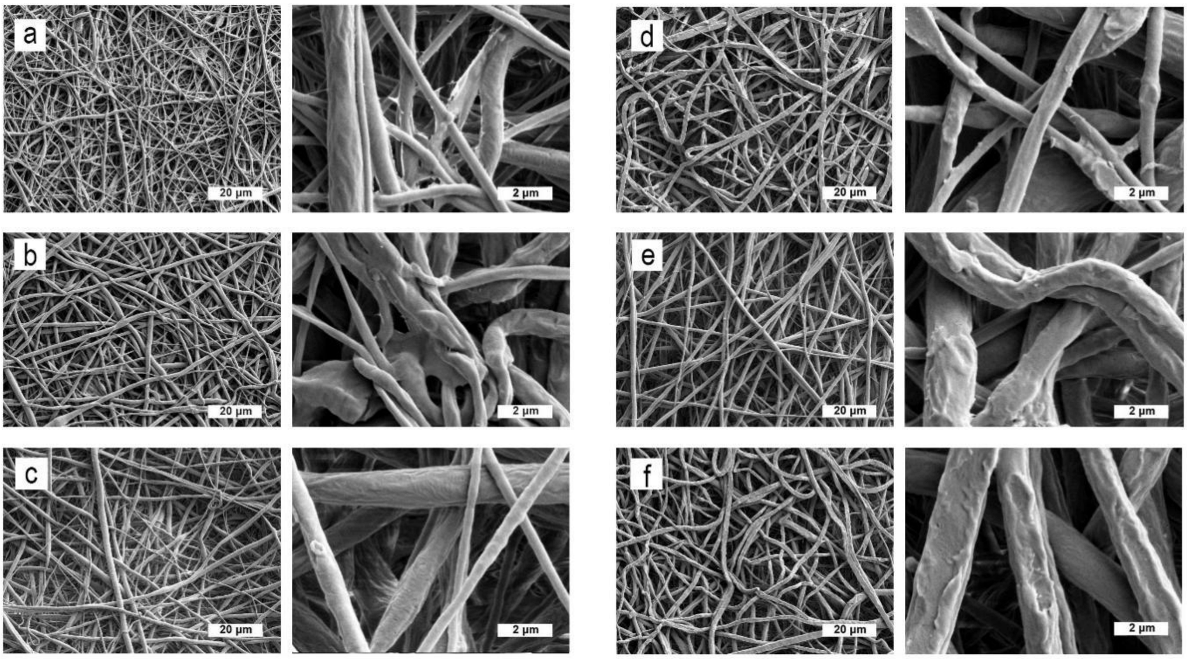

Figure S6. SEM microphotographs of magnetic polymer nanocomposites that were incubated for 368 days: (a) fPCL,(b) mfPCL, (c) mfPCL@TA-1, (d) mfPCL@TA-2, (e) mPCL@TA-5, and (f) mfPCL@TA-10.

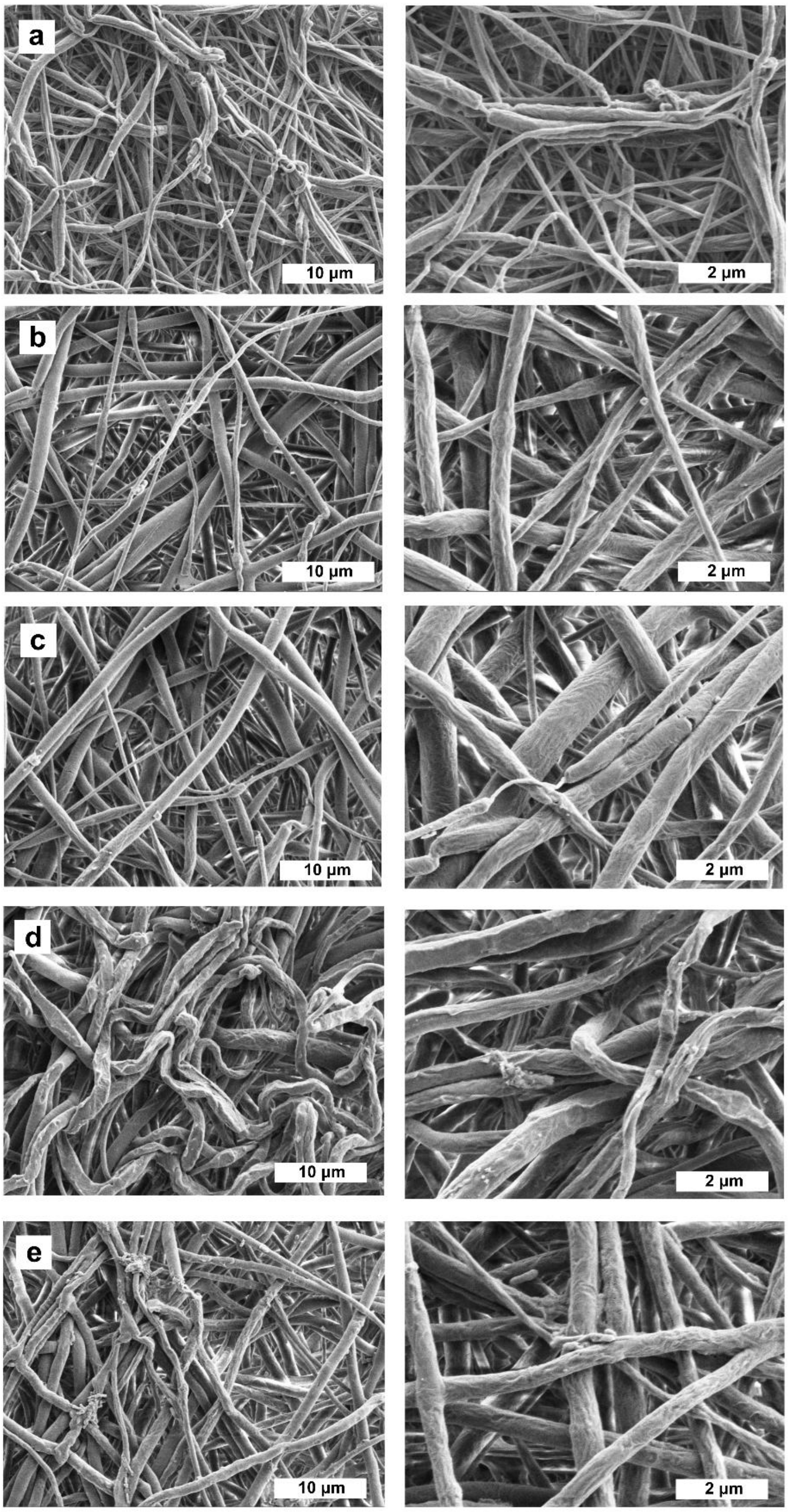

Figure S7. SEM microphotographs of magnetic polymer nanocomposites that were incubated for 718 days: (a) fPCL,(b) mfPCL, (c) mfPCL@TA-1, (d) mfPCL@TA-2, (e) mPCL@TA-5, and (f) mfPCL@TA-10.
